# Statistical Fractal Models Based on GND-PCA and Its Application on Classification of Liver Diseases

**DOI:** 10.1155/2013/656391

**Published:** 2013-10-09

**Authors:** Huiyan Jiang, Tianjiao Feng, Di Zhao, Benqiang Yang, Libo Zhang, Yenwei Chen

**Affiliations:** ^1^Software College, Northeastern University, Shenyang 110819, China; ^2^Radioactive Branch, PLA General Hospital of Shenyang Military Region, Shenyang 110016, China; ^3^Department of Information Science and Engineering, Ritsumeikan University, Shiga 5258577, Japan

## Abstract

A new method is proposed to establish the statistical fractal model for liver diseases classification. Firstly, the fractal theory is used to construct the high-order tensor, and then Generalized *N*-dimensional Principal Component Analysis (GND-PCA) is used to establish the statistical fractal model and select the feature from the region of liver; at the same time different features have different weights, and finally, Support Vector Machine Optimized Ant Colony (ACO-SVM) algorithm is used to establish the classifier for the recognition of liver disease. In order to verify the effectiveness of the proposed method, PCA eigenface method and normal SVM method are chosen as the contrast methods. The experimental results show that the proposed method can reconstruct liver volume better and improve the classification accuracy of liver diseases.

## 1. Introduction

Liver cancer is a common disease in daily life and is often diagnosed when it is advanced, and very few liver cancer patients can be cured. So it is necessary for us to diagnose liver cancer as early as possible. Computer Aided Diagnosis (CAD) technology is established with the development of computer graphics technology, image processing technology, and pattern recognition technology. Since CT images increase the burden of doctors, research of this kind of technology is urgently needed. In recent years, scientists have researched several typical variable models such as Snake Model. These methods are more suitable for objects with smooth boundary and do not use the valuable prior knowledge. Active Texture Model (ATM) which is evolved from them can reflect the texture feature of the object. The roughness of surface in medical images is an important factor to distinguish among lesions, so ATM cannot represent object features well only with the texture model of gray feature.

In order to solve the problems as above, we proposed the statistical fractal model based on the feature of gray level and fractal dimension. The statistical fractal model can be better used in the analysis of medical images such as diseases recognition, but the construction of statistical appearance model is a challenging task when the number of training samples is much fewer than the number of dimensions of data.

Principal Component Analysis (PCA) method [1] is a famous method used in the subspace recognition, and it is one of the classical methods based on statistical feature. But this method has two problems. The first is that the original space structure of image is damaged in the vectorization process. The second is that it may cause the dimension disaster when we transfer the image into a vector. So we need more large space to calculate the covariance matrix of images. In order to solve these problems, we use the Generalized *N*-dimensional PCA [2] to learn subspace in this paper.

Support Vector Machine (SVM) [3] is commonly used to train a classifier. And the factor to affect the classification performance is the parameters used in SVM. So we use Ant Colony Optimization (ACO) algorithm to optimize SVM parameters, and then we use Directed Acyclic Graph DAG [4] to multiclassify liver diseases.

As above, for protecting the special space structure information of liver images and solving the dimension disaster problem, we extracted the gray feature and fractal feature to establish the high-order tensor of liver volume and constructed the statistical fractal model with GND-PCA method. For improving recognition accuracy, SVM optimized by ACO (ACO-SVM) was used to recognize liver diseases images.

This paper is organized as follows.  Section 2 introduces the proposed method; firstly, we will introduce some knowledge of PCA and tensor, then we will show the construction of high-order tensor, and finally we introduce the method of GND-PCA for the construction of statistical fractal model and ACO-SVM [5] for classification. In Section 3, we present the construction of liver images after GND-PCA and the results of classification.  Section 4 concludes the works in this paper.

## 2. Materials and Methods

**Figure 1:**
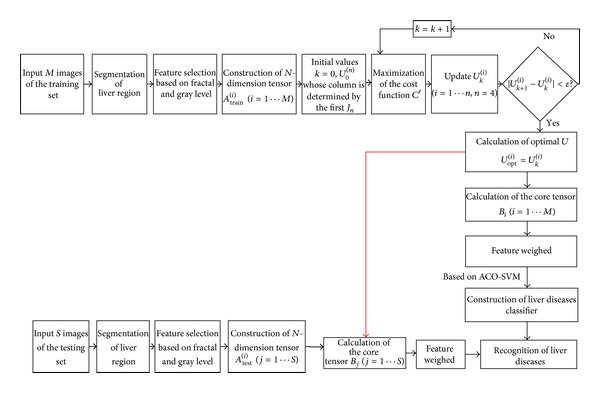
The main flow of the proposed method.

In this section, we will introduce some background knowledge about GND-PCA method firstly. We mainly present the method of PCA, 2D-PCA, and ND-PCA and the basic knowledge of tensor. And then we will introduce our method of construction of statistical fractal model. The main flow is shown in Figure 1. The process of the proposed method is described as follows.

1. Liver images preprocessing. Firstly we segment the CT images of abdomen to gain the liver region of image, and then we calculate the fractal dimension of liver image.
2. High-order tensor construction. At first, we collect a group of fractal features and gray level features (pixels), and then we combine them into a new dataset.
3. Statistical fractal model establishment. In this paper, we use the method of Generalized *N*-dimensional PCA (GND-PCA) to establish the statistical fractal model for classification.
4. Liver diseases classification based on ACO-SVM. After we obtain the statistical fractal model by GND-PCA, we treat the core tensors as samples, and then we use SVM optimized by ACO to classify liver diseases.

### 2.1. PCA Method and Its Extension

PCA is an application of *K*-*L* conversion in statistics. The purpose of PCA is to lower the dimension of data through finding a linear mapping. The mapping meets the following conditions.

1. The error of sample reconstruction is minimized.
2. The mapping of sample set in low dimension space has the maximum variance.
3. The correlation among samples is erased.

**Figure 2:**
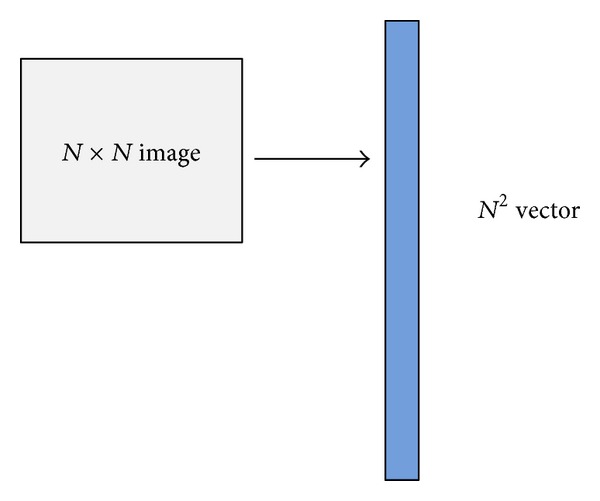
2D matrix to vector.

Turk and Pentland proposed the famous method named eigenface to realize PCA. Suppose that we have *K* training samples, *I*_1_, *I*_2_,…, *I*_*K*_. Firstly, we transfer these samples into vectors shown in Figure 2. The *N* × *N* image is transferred into a column vector; that is to say, the training samples *I*_1_, *I*_2_,…, *I*_*K*_ are transferred into *X*_1_, *X*_2_,…, *X*_*K*_. *X*_*i*_ is a column vector with the dimension of *n*  (*n* = *N* ^2^). Each *X*_*i*_ is in the space of *n* dimension. According to the knowledge of linear algebraic, *X*_*i*_ can be expressed by *n* basis in the *n* dimension space. If we express *X*_1_, *X*_2_,…, *X*_*K*_ by only one vector, obviously, we should use the average value *m* of *X*_1_, *X*_2_,…, *X*_*K*_. We rename *m* as the zero-dimension expression of sample datasets. Doing as above is useful and easy, but the shortcoming of it is that it can not show the difference between samples. So the second step of PCA is to centralize the training sample sets *I*_1_, *I*_2_,…, *I*_*K*_, and then we find the 1-dimension to *d* dimension expression of the new sample sets.

Compared with PCA, 2D-PCA uses the 2-dimension image matrix directly for feature extraction. It can calculate the covariance matrix accurately with less time. Imagining that there is an *n* dimension column vector which is normalized, we can project any image (*m* × *n*) to it, *Y* = *AX*, and get the *m* dimension image eigenvector. The separating capacity of *X* can be measured by the total divergence of the projected samples, and the total divergence of projected samples can be expressed by the trace of the covariance of the reflected eigenvector. *J*(*X*) = tr⁡(*S*_*x*_), *S*_*x*_ is the covariance of the projected eigenvector of training sample, and tr⁡(*S*_*x*_) is the trace of *S*_*x*_. The purpose of maximization of *J*(*X*) is to find the mapping direction, and the final total divergence of mapping sample is the largest. 

The advantage of 2D-PCA method is that we do not need to transform images into vectors, and we can use the images themselves directly to deal with data information and find a group of basis which can express the original samples best. Moreover, the eigenfactor is a matrix not a vector which PCA method needs. It keeps the space structure of the original images. And it does not only wipe off the correlation between the samples effectively but also wipe off the correlation between the rows in one sample. But the method has shortages too, and the mapping coefficient matrix is large and wastes lots of memory space because of the ignorance of the difference between the columns in one sample. 

Alternative 2D-PCA is proposed to overcome these problems as above. The method can solve the problem of ignorance of the difference between the columns in one sample but also cannot solve the problem of the large coefficient matrix and the difference between both columns and rows. As a result, the G2D-PCA method is proposed, and this method considers the correlation between both columns and rows. The mapping function is *C* = *Z* ^*T*^ *AX*, and it can be seen as mapping to the rows first and then to the columns or to the columns first and then to the rows. At the same time the iteration ideology is proposed by G2D-PCA to obtain better results. 

ND-PCA is proposed for modeling of high-dimension data. This method is based on HOSVD. At the same time, we treat the data as a high-dimension tensor. The method can solve the problem of high cost effectively, but it also has a large coefficient matrix as 2D-PCA method. 

### 2.2. The Basic Knowledge of Tensor

Tensor can be treated as the expansion of matrix. Vector is a first-order tensor and matrix is a second-order tensor. So if we stack up several matrixes with the same dimension, we obtain the cubic array named third-order tensor. The analysis of high-order tensor uses the math operation as follows [6].

Suppose that *X* is an *M*-order tensor, *X* ∈ *R* ^*N*_1_×*N*_2_×⋯×*N*_*M*_^, and *N*_*i*_ is the dimension of tensor *X*. The element of *X* is defined as *X*_*n*_1_,*n*_2_,…,*n*_*M*__, for 1 ≤ *n*_*i*_ ≤ *N*_*i*_, 1 ≤ *i* ≤ *M*. The tensor product is defined as follows:

(1) (X⊗Y)n1×n2×⋯×nM×n1′×n2′×⋯×nM′=Xn1×n2×⋯×nMYn1′×n2′×⋯×nM′.

We can transfer the *M*-order tensor to a matrix by extending the *N*_*d*_th vector of tensor *X* and put others after the *N*_*d*_. The product function of tensor *X* and matrix *U* is shown as follows:

(2) (X×dU)i1×i2×⋯×j×id−1×⋯×iM=∑id(Xi1×i2×⋯×id−1Uj×id).

### 2.3. Construction of High-Order Tensor

In this paper, we construct high-order tensors based on fractal theory. Firstly, we use the method of box [7] and blanked [8] to calculate 4 groups of fractal feature, and then we establish high-order tensors based on the fractal feature and the texture feature pointing to each pixel.

#### 2.3.1. The Calculation of Fractal Feature

**Figure 3:**
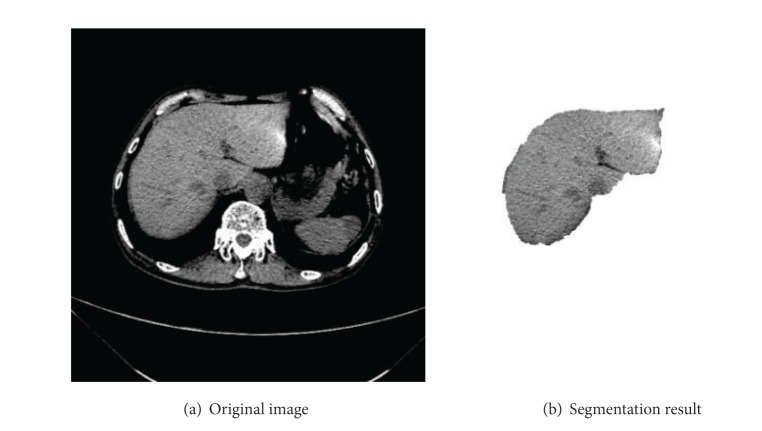
Liver segmentation.

We use the method of blanket and box to calculate the fractal feature of liver images which are segmented by the doctor. The liver image and its segmentation result are shown in Figure 3. 

The first fractal feature is obtained by the blanket method. Firstly, we treat the images as a hilly terrain surface whose height from the normal ground is proportional to the gray level of the images. Then all points at distance *ε* from the surface on both sides create a blanket whose thickness is 2*ε*. The estimated surface area is the volume of blanket divided by 2*ε*.  For different *ε*, the blanket area can be iteratively estimated as follows. The covering blanket is defined by its upper surface *u*_*ε*_ and the lower surface *d*_*ε*_, and we provide the gray level function *g*(*i*, *j*), *u*_0_(*i*, *j*) = *b*_0_(*i*, *j*) = *g*(*i*, *j*), for *ε* = 1, 2,3,…. Blanket surfaces are defined as follows:

(3) uε(i,j)=max⁡⁡{uε−1(i,j)+1,          max⁡|(m,n)−(i,j)|≤1⁡uε−1(m,n)},bε(i,j)=min⁡⁡{bε−1(i,j)−1,          min⁡|(m,n)−(i,j)|≤1⁡bε−1(m,n)}.

The volume of the blanket is defined as follows:

(4) $$\begin{array}{c} v_{\varepsilon }=\underset{i,j}{\sum }\left(u\left(i,j\right)-b_{\varepsilon }\left(i,j\right)\right). \end{array}$$

The surface area can be defined as follows:

(5) $$\begin{array}{c} A\left(\varepsilon \right)=\frac{\left(v_{\varepsilon }-v_{\varepsilon -1}\right)}{2},\\ A\left(\varepsilon \right)=F\varepsilon^{2-D}. \end{array}$$

At last, the fractal feature *D*_1_ can be described as (6), and *v* is the volume of the blanket:

(6) $$\begin{array}{c} D_{1}=2-\log_{\varepsilon }^{\left(v_{\varepsilon }-v_{\varepsilon -1}\right)/2F}. \end{array}$$

The other fractal features are obtained by the method of box. It is to treat the gray level image *F*(*R* × *R*) as a box in 3-dimensional fractal curves. The image can be separated into several boxes (*S* × *S*),  *δ* = *S*/*R*. *z* is the gray level of the images; the plane surface *XY* can be separated into several grids. The maximum level and the minimum level of gray level of the image in the grid (*i*, *j*) can be treated as the *k*th and the 1st box, *n*_*δ*_(*i*, *j*) = *l* − *k* + 1, and then we calculate the total number *N*_*δ*_(*F*) = ∑*n*_*δ*_(*i*, *j*), and the fractal feature can be defined as (7). In this paper, we obtain *D*_2_ to *D*_4_ by giving different numbers of the boxes such as 4, 8, and 16:

(7) $$\begin{array}{c} D_{B}=\frac{\log N_{\delta }\left(F\right)}{\log\left(1/\delta \right)}. \end{array}$$

There is a big texture difference in coarse level between different liver images, but the fractal dimension has a small change in smooth-faced images and a large change in shaggy images. So the fractal dimension is a useful feature for liver diseases classification.

#### 2.3.2. The Construction of High-Order Tensor

In this paper, we use 50 groups of liver images of 512 × 512. After we extract four kinds of fractal features, we extract the texture features. We use all features we obtained to establish the high-order tensors.

### 2.4. The Construction of Statistical Fractal Dimension Based on GND-PCA

**Figure 4:**
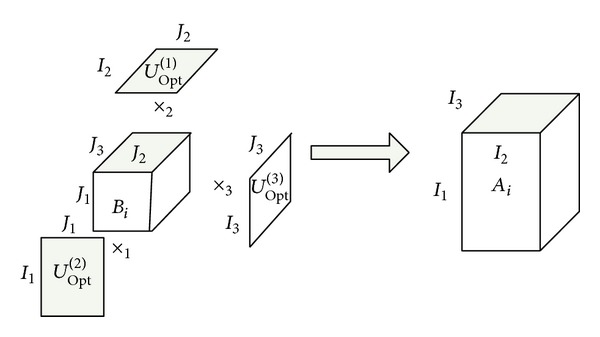
Reconstruction of third-order tensor image.

We provide a series of zero-mean value *N*-order tensor *A* ∈ *R* ^*I*_1_×*I*_2_×⋯×*I*_*N*_^. And we need to gain a group of new *N*-order tensor *B* ∈ *R* ^*J*_1_×*J*_2_×⋯×*J*_*N*_^(*J*_*n*_ < *I*_*n*_), and *B* needs to be closed to the original tensor as much as possible. Then we define tensor images by the texture and fractal features obtained from the segmented liver images. We use Tucker model [9] to reconstruct *N*-order tensor *A* by *U*_(*n*)_, *U* ^(*n*)^ = *J*_*n*_ × *I*_*n*_. The reconstruction of three-order tensor is shown in Figure 4.

The orthogonal matrix *U*_opt_^(*n*)^ can be obtained by minimizing the cost function *C*, which is shown as (8).

In *A*_*i*_ ∈ *R* ^*I*_1_×*I*_2_×···×*I*_*N*_^, *i* = 1,2,…, *M*, *M* is the number of samples. *A*_i_* is the reconstructed tensor. There are two methods to minimize the cost function:

(8) C=∑i=1M||Ai−Ai∗||2=∑i=1M||Ai−Bi×1U(1)×2U(2)×⋯×NU(N)||2,

(9) $$\begin{array}{c} A_{i}^{*}=B_{i}\times_{1}U^{\left(1\right)}\times_{2}U^{\left(2\right)}\times \cdots \times_{N}U^{\left(N\right)}. \end{array}$$

The first is to minimize the cost function *C* directly, and we can calculate the orthogonal matrix by the function *B*_*i*_ = *A*_*i*_×_1_ *U* ^(1)^ ^*T*^×_2_ *U* ^(2)^ ^*T*^ × ⋯×_*N*_ *U* ^(*N*)^ ^*T*^. But it is difficult to calculate the function. The second method is to maximize *C*′ shown as (10), and it is easier to calculate. In this paper, we used the second method:

(10) $$\begin{array}{c} C^{\prime }=\overset{M}{\underset{i=1}{\sum }}{||A_{i}\times_{1}{U^{\left(1\right)}}^{T}\times_{2}{U^{\left(2\right)}}^{T}\times \cdots \times_{N}{U^{\left(N\right)}}^{T}||}^{2}. \end{array}$$

### 2.5. Construction of the Classification of Liver Diseases

In this paper, ACO is used to optimize SVM to train a liver diseases classifier. DAG structure for multiclassification is used to distinguish liver diseases. 

#### 2.5.1. Feature Selection Based on Liver Statistical Fractal Model

The samples consist of the core tensor of each tensor. We transfer the core tensor into a one-dimensional vector using the method of nonlinear data dimensionality reduction [10]. The training set is *D* = {*d*_1_, *d*_2_,…, *d*_*M*_} (*M* is the total number of the samples), and the set of features is *T* = {*t*_1_, *t*_2_,…, *t*_*P*_} (*P* is the total number of the features).

#### 2.5.2. Feature Weighed

The number of gray level features we select is too much, and the number of fractal features is fewer than it. So we give a higher weight to the fractal features. A series of experiments showed that the classification accuracy is much better when the weight of fractal feature is 0.6 and the weight of gray level feature is 0.4.

#### 2.5.3. Construction of Classifier Based on ACO-SVM

**Figure 5:**
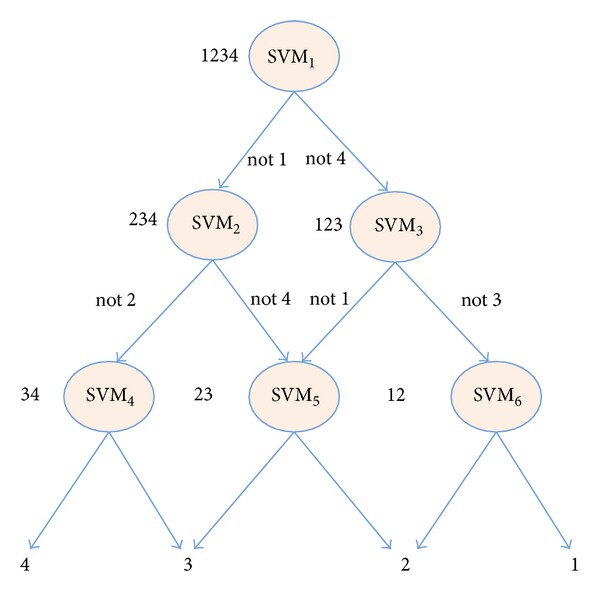
DAG-SVM, *k* = 4.

Some diseases such as cirrhosis and hepatic cyst are different from cancer. They are usually confused with cancer in CAD. SVM is always used in binary classification. If we want to classify 4 kinds of liver diseases using SVM, we should combine several SVMs. In this paper we use the method of directed acyclic graph (DAG-SVM (*k* = 4)) to realize the multiclassification of liver diseases, and DAG is shown in Figure 5.

If we classify 4 kinds of liver diseases, we should use 6 SVMs. *C* is the penalty factor, and *σ* is the parameter of kernel function. In order to optimize these two parameters by ACO algorithm, *C* and *σ* must be discretized firstly. In this paper, the two parameters are discretized according to effective bits which are determined by experiences. The parameter *C* and *σ* has five effective bits, respectively. The value of each bit can be varied from 0 to 9. For *C*, its top digit is hundreds place, so its value ranges from 0 to 999.99. While for *σ*, its top digit is ones place, and thus its value ranges from 0 to 9.9999.

Then heuristic information *η*(*i*, *j*) is set to 1. Classification accuracy is used to evaluate SVM performance, and therefore Δ*τ*(*i*, *j*) = *Q* · Acc is used in the global update process. Here *Q* is pheromone intensity and Acc is maximal classification accuracy in each cycle. The whole process is executed as follows.

**Step 1**

Discretizing parameters *C* and *σ* by the method as above.

**Step 2**

Initializing pheromone *τ*(*i*, *j*) = 1 and pheromone increment Δ*τ*(*i*, *j*) = 0.

**Step 3**

Executing search process for the first best path.

1. Laying ants at the origin of coordinates.
2. Putting each ant to next city whose *x* coordinate is different from the previous visited cities randomly.
3. Modifying pheromone of transfer path for each ant according to local update rule.
4. Modifying pheromone of the path for the best ant according to global update rule if all the ants finish visiting 10 nodes, else returning to (2).

**Step 4**

Laying ants at coordinate origin again.

**Step 5**

Putting each ant to the next city chosen according to state transition rule.

**Step 6**

Modifying pheromone of transfer path for each ant according to local update rule. If ants finish tour, we jump to Step 7; otherwise we return to Step 5.

**Step 7**

Training a SVM classifier with *C* and *σ* obtained by each ant. We find out the best ant which produced the highest accuracy and modify pheromone for the best ant according to global update rule. If the accuracy meets termination condition or the times of loop are bigger than the maximum cycle times, we jump to Step 8; otherwise we return to Step 4.

**Step 8**

Outputting best *C*, *σ* and maximum accuracy.

## 3. Results and Discussion

We select 120 groups of liver images, 60 groups are normal liver, 20 groups are cirrhosis liver volume, 20 groups are cancer liver volume, and 20 groups are hydatoncus liver volume. There are 50 images in each group. The thickness of each image is 3 mm, and the resolution is 512 × 512. In 120 groups of images, we selected a half as training samples, the others as testing samples.

### 3.1. Reconstruction Results after GND-PCA

**Figure 6:**
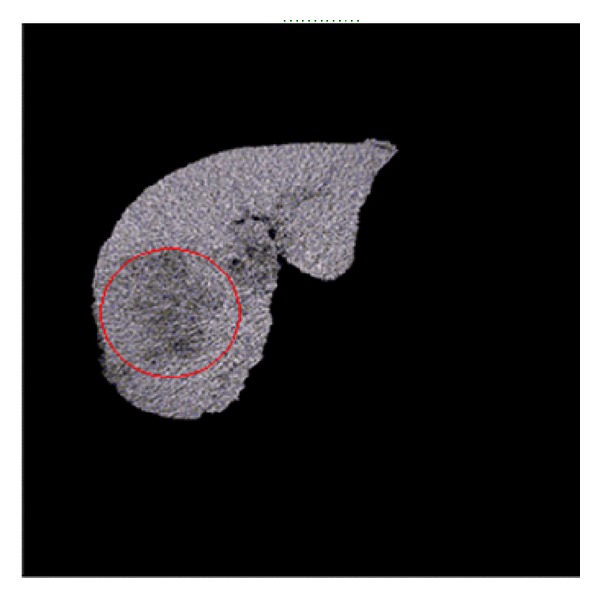
Liver cancer image.

In this paper, we use leave-one-out method to test the generalization ability of models constructed without fractal features for liver volumes. One of all images is shown in Figure 6. The location of tumor is in the lower left corner of the liver image. Firstly, one volume is excluded from the training data which is used for the construction of the model, and then it is reconstructed by the training models for checking.

**Figure 7:**
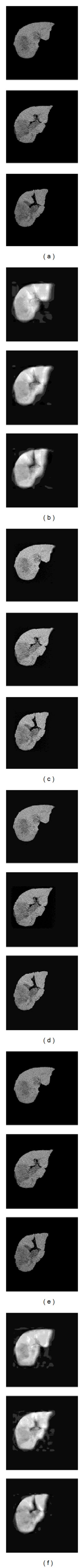
Reconstruction results.

The volume is reconstructed from 5 × 5 × 3 to 300 × 300 × 30 which is shown in Figure 7. In Figure 7, the first row is reconstruction of slice 3, the second row is reconstruction of slice 13, and the third row is reconstruction of slice 23. Column (a) is original liver image, column (b) is that the dimension of mode-subspace is 5 × 5 × 3, column (c) is 100 × 100 × 10, column (d) is 200 × 200 × 20, column (e) is 300 × 300 × 30, and column (f) is the reconstructed volume using eigenface by PCA as the contrastive method. 

Since the dimension of the original volume is 512 × 512 × 50, we can calculate the compressing rate for all cases. The compressing rate is 0.0006%, 0.7629%, 6.1035%, and 20.5994%. With the growth of the dimension of mode-subspace, reconstruction result is better. Because of overfitting, the method of PCA is worse than GND-PCA.

**Figure 8:**
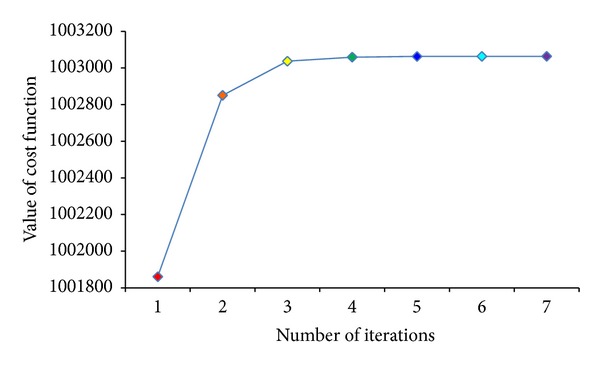
Convergence of GND-PCA.

It needs less iteration times using GND-PCA which is shown in Figure 8, and the value of the cost function does not dramatically change after two iterations. Therefore, we set the iteration times of GND-PCA as two in our experiment.

**Figure 9:**
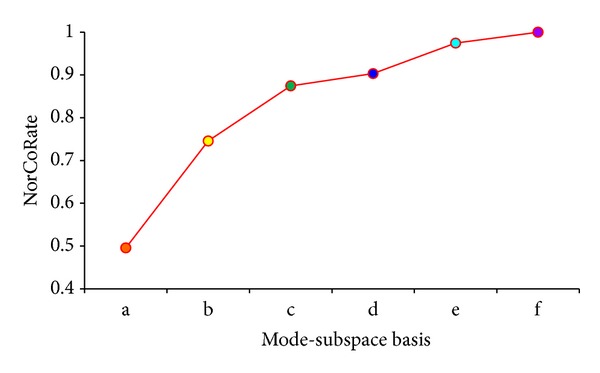
Normalized correlation between the original volume and the reconstructed volumes.

In Figure 9, it shows the relationship between original volume and the reconstructed volume. Abscissa a is the mode-subspace of 5 × 5 × 3, b is 100 × 100 × 10, c is 200 × 200 × 20, d is 300 × 300 × 30, e is 400 × 400 × 40, and f is 512 × 512 × 50.

The normalized correlation grows with the growth of mode-subspace size. When the mode-subspace size is 512 × 512 × 50, the normalized correlation is 1. It means that we can reconstruct the original volume without any errors. The normalized correlation can be defined as (11). *I*(*x*, *y*, *z*) is the original tensor volume, and I*(*x*, *y*, *z*) is the volume after reconstruction:

(11) $$\begin{array}{c} NC=\frac{\sum_{x,y,z}I\left(x,y,z\right)I^{*}\left(x,y,z\right)}{\sqrt{\sum_{x,y,z}I^{2}\left(x,y,z\right)}\sqrt{\sum_{x,y,z}I^{*2}\left(x,y,z\right)}}. \end{array}$$

### 3.2. Results of Classification

The result of each SVM in ACO-SVM multiclassifier is shown in Table 1. From the table we can see that the statistical fractal model has better accuracy than the statistical texture model without fractal feature.

**Figure 10:**
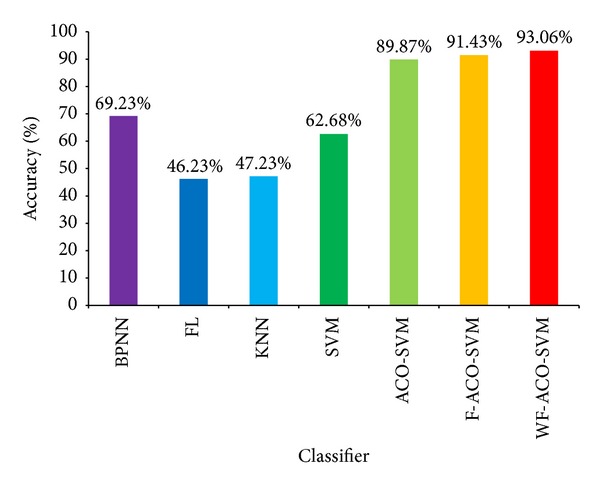
Result of multiclassification using seven classifiers.

Compared with other classifier, ACO-SVM with the weighed fractal feature has better accuracy which is shown in Figure 10. Classifier BPNN is BP neural network, and the accuracy is 69.23%. Classifier FL is Fisher linear classifier with 46.23% accuracy. Classifier KNN is k-Nearest Neighbor algorithm whose accuracy is 47.23%. Classifier SVM is the conventional SVM with 62.68% accuracy. Classifier ACO-SVM is the conventional ACO-SVM whose accuracy is 89.87%. Classifier F-ACO-SVM is the method which is ACO-SVM with fractal features, and the accuracy is 91.43%. Classifier WF-ACO-SVM is ACO-SVM with weighed fractal feature; the accuracy is 93.06%. As Figure 10 shows, ACO-SVM does better than others in classification. And when we use weighed fractal feature in our statistical fractal model, we can reach a better accuracy in liver diseases classification.

## 4. Conclusions

In this paper, we have presented the construction of high-order tensors with weighed fractal dimension feature and gray feature. And GND-PCA, which is a subspace learning method, has been used to get the core tensor from those high-order tensors and establish the statistical fractal model for the later classification. ACO-SVM has been used to train a liver image classifier. As an application for classifying liver diseases, the method using statistical fractal models based on GND-PCA and ACO-SVM achieved the better classification accuracy, because statistical fractal models based on GND-PCA can preserve the information of the original image as much as possible, and ACO can find the optimal parameters for SVM. In conclusion, under the condition of a small number of samples, the classifier of this paper can achieve the better recognition accuracy than others such as BPNN, the conventional SVM, and the conventional ACO-SVM. Therefore the proposed method can improve the classification accuracy of liver diseases and assist doctors to diagnose liver diseases.

## Figures and Tables

**Table 1:** Parameters optimization result of SVM for multiclassification using ACO.

Classifier	Best *C *	Best *δ*	NFD Acc	FD Acc	WFD Acc
ACO-SVM_1_	622.57	1.3114	96.44%	97.64%	97.85%
ACO-SVM_2_	783.96	5.2349	98.02%	98.52%	98.64%
ACO-SVM_3_	100.230	1.2255	97.74%	99.87%	99.87%
ACO-SVM_4_	14.020	0.2378	99.76%	99.83%	99.98%
ACO-SVM_5_	984.69	1.1424	94.3%	96.57%	96.65%
ACO-SVM_6_	876.78	1.0765	98.82%	99.33%	99.64%
